# Effects of a Gastroscopic Procedure on Salivary Cortisol Release and Fecal Cortisol Metabolites in Young Racehorses

**DOI:** 10.3390/ani14223332

**Published:** 2024-11-19

**Authors:** Sabina Ostermeier, Rupert Palme, Ingrid Vervuert, Barbara Glomm, Karsten Feige, Sabine Macho-Maschler, Uta König von Borstel, Monica Venner

**Affiliations:** 1 Equine Clinic, University of Veterinary Medicine Hanover, 30559 Hanover, Germany; sabinaostermeier1@gmail.com (S.O.); karsten.feige@tiho-hannover.de (K.F.); 2 Experimental Endocrinology, Department of Biological Sciences and Pathobiology, University of Veterinary Medicine, 1210 Vienna, Austria; rupert.palme@vetmeduni.ac.at (R.P.); sabine.macho-maschler@vetmeduni.ac.at (S.M.-M.); 3 Institute of Animal Nutrition, Nutrition Diseases and Dietetics, Leipzig University, 04109 Leipzig, Germany; barbara.mockenhaupt@gmx.de; 4 Institute of Animal Breeding and Genetics, Justus-Liebig-University Giessen, 35392 Giessen, Germany; uta.koenig@agrar.uni-giessen.de; 5 Equine Clinic Destedt GmbH, Destedt, 38162 Cremlingen, Germany; mvenner@gmx.de

**Keywords:** gastroscopy, stress, salivary cortisol, fecal glucocorticoid metabolites, Thoroughbred racehorses

## Abstract

In this study, gastroscopy was performed twice in 31 two-year-old Thoroughbred racehorses in training. Salivary cortisol (SC) and fecal cortisol metabolite (FCM) concentrations were determined to measure the stress impact. One gastroscopy was performed at the beginning and one six months later at the end of the horses’ first training season. According to the Wilcoxon signed rank test, a mild but significant increase of SC (1st gastroscopy: *p* = 0.0045, 2nd gastroscopy: *p* < 0.0001) and FCM (1st gastroscopy: *p* < 0.0001, 2nd gastroscopy: *p* = 0.0006) values after gastroscopy compared to basal values was detected.

## Simple Summary

Gastroscopy is a minimally invasive, safe method to evaluate the equine stomach. This procedure is important for a precise diagnosis in case of suspected gastric disease for the adequate treatment and management of equine patients. However, horses might experience stress while undergoing gastroscopy. In this study, a moderate salivary cortisol release and a mild increase in fecal cortisol metabolites were detected in two-year-old Thoroughbred racehorses after gastroscopy. However, in comparison to other stressful situations, such as transportation, gastroscopy performed in the horse’s familiar environment can be considered a minor stress event.

## 1. Introduction

Gastric diseases in horses are frequent and patients often show only unspecific signs. Therefore, gastric disorders can be challenging to diagnose in equine practice [1]. Not only lesions of the gastric mucosa but also gastric impaction or neoplasia and inflammation can lead to clinical problems [2]. So far, gastroscopy is the only method that allows a detailed visualization of all regions of the stomach, necessary for a precise diagnosis [3]. Moreover, gastroscopy is considered to be a minimally invasive, safe method to evaluate the equine stomach [4]. Knowledge about equine gastric ulcer syndrome (EGUS) is increasing and its prevalence, causes and treatment strategies are becoming well identified [5]. Consequently, it appears crucial to differentiate between equine squamous gastric disease and equine glandular gastric disease (EGGD) [5,6]. Equine squamous gastric disease, EGGD and their specific lesion types and severity are related to well-defined anatomical regions. The term EGUS should primarily be used as a general term for erosive and ulcerative stomach disease, as suggested in the latest European College of Equine Internal Medicine Consensus Statement. Precise diagnosis of the different lesion types via gastroscopy is, thus, the key to successful patient management, because the pathomechanisms and treatment of ESGD and EGGD differ substantially [4].

Consequently, gastroscopy is commonly used in equine practice, even if it might cause stress to the horses. Stress has recently been brought up as a risk factor especially for EGGD [7,8]; thus, it appears worthwhile to investigate whether a horse experiences stress during gastroscopy. Stress and its potential negative impact on suspected EGUS is also one of the biggest concerns of horse owners regarding the gastroscopic procedure. This often leads to rejections of gastroscopy and, accordingly, frequently to unsuccessful treatment.

Stressful situations are known to stimulate the hypothalamic–pituitary–adrenal axis followed by a release of corticotropin releasing hormone. The latter increases adrenocorticotropic hormone production by the pituitary gland, which finally stimulates cortisol release from the adrenal cortex [9]. The measurement of glucocorticoids to monitor hypothalamic–pituitary–adrenal activity is a standard procedure in studies evaluating stress and well-being in horses and other animals [10,11]. There are different methods to measure glucocorticoids. Although measuring glucocorticoids in blood was the gold standard for a long time, alternative methods, such as cortisol determination in saliva or cortisol metabolite determination in feces, have become more attractive. By being less invasive, these methods cause less additional stress for the animal than blood collection [11,12].

Glucocorticoids secreted by the hypothalamic–pituitary–adrenal axis are primarily metabolized by the liver and excreted predominantly as metabolites via bile [11,13]. Fecal cortisol metabolites (FCMs) are detectable in the adult horse after its species-specific time delay of 24 h, roughly corresponding with the horse’s gastrointestinal passage time [14]. Both FCMs and salivary cortisol (SC) reflect the unbound plasma cortisol [15]. Furthermore, FCMs generally reflect more chronic stressors, while SC increases soon after acute stress [16,17]. The current study was designed to evaluate the impact of a gastroscopic procedure on stress-related cortisol release in saliva and feces in young racehorses in training.

## 2. Materials and Methods

### 2.1. Animals and Experimental Design

The study included 36 two-year-old, privately owned Thoroughbred horses (23 mares, 13 stallions) from five different training stables in Germany (Trainer A–E), in active training for flat racing with a licensed trainer. At the first gastroscopy, the median age of the horses was 26 months (min. 20 months, max. 28 months) and on average the training was up to 30–40 km/h. At the second gastroscopy the age was 32 months (min. 26 months, max. 34 months) and on average the training was up to 45–50 km/h. All the horses were housed in individual boxes on straw bedding, some with daily paddock turnout alone (Trainer A) or in small groups (Trainer B, C, E) on pasture. The horses of Trainer D only had irregular paddock turnout less than twice a week. All the horses received meadow hay ad libitum. In addition, all the horses were fed with oats and concentrate feed. They had access to a salt block. Some horses were supplemented with soybean extract, flax and sunflower seeds, commercial mineral feed, electrolytes or oil. The study was authorized by the state ethical committee (LAVES AZ: 33.19-42502-04-22-00041). Owners and trainers gave permission for study enrollment. In addition, informed consent was obtained from all animal owners involved in the study.

The study consisted of two identical periods, one at the start of the horses’ first race-season (May 2022) and the other six month later (November 2022). In the second period, only 31 of the former 36 horses participated (20 mares, nine stallions, two geldings). Two stallions were neutered between the study periods. Five horses were withdrawn from the study due to a change of trainer.

Each study period included the collection of basic data (age in months, sex, body weight and body condition according to the scoring system (score 0–5) of Carroll and Huntington [18]), trainer, training status, type/number of daily meals). Gastroscopy was performed identically in both study periods. Three saliva samples were collected prior to gastroscopy in order to determine baseline SC on three consecutive days (days 1–3) at the same time of day. Fourth and fifth saliva samples were collected on day 4 immediately after and 30 min after, respectively, finishing the gastroscopy. Fecal samples were collected from each horse at the same time of the day on days 2, 3 and 4 for basal value determination of FCMs and a fourth sample was collected 24 h after each gastroscopy (Table 1).

The horses’ overall health was evaluated by two veterinarians prior to each gastroscopy by clinical examination. In addition, full blood work including hematology and serum biochemistry (Equine Clinic, University of Veterinary Medicine Hanover, Inhouse Labor, Germany) was performed in both study periods. Accordingly, blood was drawn from the jugular vein via the same venipuncture initiating sedation with detomidine (0.01–0.02 mg/kg; Cepesedan 10 mg/mL, cp-pharma, Burgdorf, Germany) for the gastroscopy. A single-use 20 G injection cannula (Sterican, Braun-Melsungen AG, Melsungen, Germany) and a 20 mL syringe (Henke-ject 20 (22) mL, Henke Sass Wolf, Tuttlingen, Germany) were used to draw blood from the vein. The blood was transferred into an EDTA tube and lithium heparin tube (Li-Heparin LH/4.5 mL, EDTA KE/3 mL, SARSTEDT AG & Co. KG, Sarstedt, Germany) for further analysis. The laboratory-specific reference ranges from Rossdales Laboratories for two-year-old Thoroughbred horses in training were used for the evaluation of the complete blood count and serum biochemistry values obtained from the horses in the current study [19].

### 2.2. Gastroscopy

The horses fasted for 12 h wearing a muzzle prior to gastroscopy and deprived of water for the final 2 h. After sedation with detomidine (0.01–0.02 mg/kg; Cepesedan 10 mg/mL, cp-pharma, Burgdorf, Germany) a flexible three-meter video endoscope (60130 PKS/NKS (Karl Storz GmbH & Co. KG, Tuttlingen, Germany) was inserted via the ventral nasal passages and the esophagus into the stomach. Air was insufflated until the gastric mucosa straightened out. All stomach regions (saccus caecus, squamous mucosa, glandular mucosa, antrum, pylorus) were examined. Videos and photos were frequently taken of the different stomach regions. The air was withdrawn via an aspiration pump. The whole examination lasted approximately 10–15 min per horse. Gastroscopies were performed using a transportable system, allowing the horses to stay in their boxes for the whole procedure, without leaving their familiar environment. One person familiar to the horses fixed each horse and the nose twitch during the procedure, another person introduced the gastroscope via the ventral meatus, and a third person steered the gastroscope, and insufflated and withdrew the air.

Only one of the horses (as a 5-month-old foal) had previously undergone a gastroscopic procedure before the study. The rest of the horses were not familiar with this kind of procedure during the first study period.

### 2.3. Sampling of Saliva and Method for Measuring the Salivary Cortisol Concentration

Five samples of saliva were collected each in the first and second study periods in exactly the same manner. Saliva was collected with specific cotton rolls (Salivette, Sarstedt, Nümbrecht, Germany) placed under the tongue with long forceps for at least one minute until fully soaked. Collection was tolerated without any resistance by the horses. The saliva-soaked cotton was then placed into a polypropylene Salivette tube and immediately centrifuged at 1000× *g* for 10 min. At least 0.5 mL of saliva per sample was obtained, frozen and stored at −20 °C in a transportable freezer. The cortisol concentration was determined directly without extraction using a cortisol enzyme immunoassay [16] validated for horses [20]. Because the antibody cross-reacts with cortisol metabolites including cortisone, values represent “cortisol immunoreactivity”. The intra- and inter-assay coefficient of variation was below 10 %, and the minimum detectable concentration was 0.1 ng/mL.

The basal and gastroscopy SC concentrations of seven horses are missing in the first study period due to logistic problems. Furthermore, the results of the SC concentrations at 30 min after gastroscopy are missing for two horses in the first study period. The results for the gastroscopy SC concentration and the SC concentration at 30 min after gastroscopy for one horse is missing in the second study period.

### 2.4. Sampling of Feces and Method for Measuring the Fecal Cortisol Metabolites

Four samples of feces were collected each in the first and the second study period in exactly the same manner. Only fresh feces, immediately after defecation, were collected (feces tube, Sarstedt AG & Co. KG, Sarstedt, Germany) to avoid falsely high concentrations as a result of potential further metabolism by bacterial enzymes. The samples were immediately stored at −20 °C in a transportable freezer until further analysis [21]. Firstly, 0.5 g of each wet fecal sample were suspended in 5 mL of 80% methanol. Because low FCM concentrations occur in the feces of horses, a further extraction step with diethyl ether was necessary [22]. Following extraction [23], FCMs were determined using an 11-oxoetiocholanolone enzyme immunoassay [16], which measures 11,17-dioxoandrostanes, a group of cortisol metabolites. This EIA has previously been successfully validated for horses [23]. The intra- and inter-assay coefficient of variation was below 10%, and the minimum detectable concentration was 0.1 ng/g.

### 2.5. Statistical Analysis

SAS^®^ statistical software, version 9.4M7 with SAS Studio Enterprise, Version 3.8.2 (SAS Institute Inc., Cary, NC, USA) was used for the statistical analysis.

According to the Shapiro–Wilk test SC, FCM, blood values and general examination parameters did not show a normal distribution. Therefore, median and 25th and 75th percentile were used for descriptive statistics. The Wilcoxon signed rank test was used to test for differences between the gastroscopy-associated and basal SC and FCM values in each study period separately. The significance level was set at *p* < 0.05.

## 3. Results

### 3.1. Clinical Examination

Clinical parameters were within the normal range in all horses in both study periods (see Table 2). None of the horses had to be excluded due to signs of illness. A mildly enlarged, but not painful, left mandibular lymph node was noticed in two horses while being examined in the second study period.

### 3.2. Blood Values

Leukocyte values were within the normal range in all horses. Erythrocyte numbers, packed cell volumes and hemoglobin concentrations were within normal limits in almost all horses. Mild elevations of these parameters were observed in five horses in the first study period (packed cell volume: 0.491 L/L, 0.499 L/L and 0.473 L/L; hemoglobin: twice 17.5 g/dL).

Two horses showed increased serum glutamate dehydrogenase activity (46.2 and 22.6 IU/L) during the first study period. An increased serum aspartate aminotransferase activity was found in one horse (1058 IU/L), and another horse showed a mildly elevated gamma-glutamyl transferase (46 IU/L) activity, while in the second study period, glutamate dehydrogenase and aspartate aminotransferase were above the upper reference range in one horse (glutamate dehydrogenase: 15.4 IU/L; aspartate aminotransferase: 1545 IU/L). The gamma-glutamyl transferase (41 IU/L and 61 IU/L) was elevated in two horses, and serum creatinine kinase (932 IU/L) in one horse (see Supplementary Materials Table S1).

### 3.3. Salivary Cortisol Concentrations

The median and percentile SC baseline values were similar between the first period (1.8, percentiles 1.1–3.6 ng/mL) and the second study period (1.8, percentiles 1–2.3, Table 3). The median gastroscopy SC concentrations increased from 3.4 ng/mL (percentiles 2.3–4.5 ng/mL) in the first study period to 3.7 ng/mL (percentiles 1.9–6 ng/mL) in the second study period. The median SC concentrations at 30 min after gastroscopy decreased from 3.2 ng/mL (percentiles 2.7–3.9 ng/mL) in the first study period to 2.7 ng/mL (percentiles 1.5–3.9 ng/mL) in the second study period.

The SC concentrations increased significantly from the basal values (Table 3) to the gastroscopy values (1st study period *p* = 0.0045, 2nd study period *p* < 0.0001). There was a decrease in both study periods between the gastroscopy SC values and the SC concentrations at 30 min after gastroscopy (Table 3), but the decrease was statistically significant only in the second study period (*p* = 0.0008; 1st study period *p* = 0.49).

### 3.4. Fecal Cortisol Metabolites

The median basal FCM concentration was 9.4 ng/g (percentiles 7.9–11.2 ng/g) in the first study period (Table 4) and decreased to a median concentration of 7.8 ng/g (percentiles 6.4–9.1 ng/g) in the second study period (Table 4). The median gastroscopy FCM value was 12.1 ng/g (percentiles 9.6–16.3 ng/g in the first study period and 10.1 ng/g (percentiles 6.8–15.9 ng/g) in the second study period. The gastroscopy FCM values increased significantly in both study periods in comparison to the basal values (1st study period *p* < 0.0001, 2nd study period *p* = 0.0006).

## 4. Discussion

Stress can have multifactorial origins in horses. Stress per se is not inevitably harmful as a physiological mechanism [24]. However, stress is frequently discussed as a disease-causing agent in equine medicine today, especially in EGGD [7,8]. Many horse owners misleadingly associate the origin of gastric disease only with stress and therefore think that gastroscopy along with its inevitable feed and water deprivation has a negative impact on EGUS. Stress is only one single risk factor for EGUS in addition to many other factors [4]. The study clearly showed that the SC and FCM levels increased mildly after gastroscopy in both examination periods in two-year-old Thoroughbred racehorses. However, in comparison with other situations, the stress response to gastroscopy seemed moderate. German Warmbloods, for instance, showed cortisol values up to 5.7 ng/mL in the saliva and FCM values up to 95.2 ng/g in their feces after being transported for one hour [25]. In the same study, maximal values of SC and FCMs were reached 8 h after transportation [25]. Merl et al. investigated FCM levels of Haflinger stallions after castration at their home barn, measuring the highest values two days after surgery with 19.7 ng/g [22]. They also demonstrated that there is a positive correlation between colic severity and FCM values, with peak concentrations up to 79 ng/g [22]. Both studies cited used the same assay as in the present study. Therefore, the SC and FCM values should be comparable.

Malmkvist et al. showed that Danish Warmbloods had an increase of FCMs after being confronted with a novel object [23]. The first gastroscopy for the two-year-old Thoroughbred racehorses was also a new procedure in the first study period for all horses but one. Although the second gastroscopy represented a repetition for the study horses, the rise in the SC and FCM concentrations after gastroscopy was similar in both study periods. Only the decrease of the SC from immediately after gastroscopy to the concentration at 30 min after gastroscopy was higher in the second than in the first study period, showing statistical significance in the second study period. This result suggests that the horses might have been able to cope more easily with a repeated gastroscopic examination.

The horses participating in the study remained in their familiar environment for the period of feed deprivation (12 h) as well as for the whole gastroscopy procedure. Horses that fasted and were examined in equine clinics would have to be transported to certain facilities along with being in an unfamiliar environment, and this accumulation of stressors could lead to an overall higher stress level and thus higher SC and FCM concentrations. This possibility would have to be evaluated in a future study.

Because of the lack of specific scientific data regarding cortisol release in fasting horses, it is not known how much cortisol release was caused due to gastroscopy per se or due to the fasting period, wearing a muzzle and venipuncture in the current study. The inclusion of a control group of horses that underwent the fasting period with muzzling and venipuncture, but without gastroscopy, would have made it possible to evaluate the influence of gastroscopy alone. Another limitation is the lack of a physiological reexamination after gastroscopy with missing vital parameters. Stress leads to higher heart and respiratory rates as well as to a higher body temperature [26]. Accordingly, besides increased SC and FCMs following gastroscopy, measuring heart rate, respiratory rate and body temperature could have given additional information of the horses’ stress response. However, sedation could have led to a lower heart rate as detomidine decreases the heart rate for at least 45 min [27]. Another additional parameter could have been serum cortisol. Peeters et al. found a positive, significant correlation between serum cortisol and salivary cortisol (*p* < 0.001) [28], while Pell and McGreevy et al. were only able to observe this correlation in horses with an oral stereotypy [29]. SC, besides being pain-free and less invasive for the horse, also represents free, unbound cortisol. This biologically active free cortisol is a more sensitive parameter for hypothalamic–pituitary–adrenal activity monitoring [30]. Schneidegger et al. and Peeters et al. demonstrated with an ACTH administration that measuring SC is a non-invasive technique for assessment of adrenocortical activity in horses [28,31]. In addition, Bohak et al. found a circadian rhythm of SC in horses [32]. The gastroscopy of each horse was not exactly at the same time of day. Thus, an influence of the circadian rhythm might have been present. Irvine et al. confirmed that a circadian cortisol pattern can be easily disrupted by minor changes in the horse’s environment, management or daily routine [33]. Nevertheless, all gastroscopies were performed in the time frame between 8:00 and 10:30. Thus, no huge influence of a possible diurnal rhythm was expected. The main limitation of this study is that the 31 horses were housed in five different facilities with different training, husbandry and feeding management.

## 5. Conclusions

The study evaluated the stress response associated with gastroscopy in young racehorses, using salivary cortisol (SC) and fecal cortisol metabolites (FCMs) as indicators. Results showed mild but significant increases in SC and FCM levels following gastroscopy, suggesting a moderate stress response compared to other stress-inducing situations. The significant decrease of SC 30 min after the second gastroscopy indicates that the effect of the stressor was only acute. While the study provided valuable insights, limitations included the absence of a control group to disentangle the impact of fasting and venipuncture from gastroscopy-related stress and variability due to differing environments and management practices. Future studies should consider standardized conditions and include a control group to more accurately isolate stressors associated with the procedure of gastroscopy.

## Figures and Tables

**Table 1:** Time schedule and sampling during each study period.

Day 1	Day 2	Day 3	Day 4	Day 5
10:00 AM 1st basal SC sample	10:00 AM 2nd basal SC sample + 1st basal FCM sample	10:00 AM 3rd basal SC sample + 2nd basal FCM sample	08:00 AM Gastroscopy and gastroscopy SC sample + 30 min after the end of each horse’s gastroscopy SC sample 10:00 AM 3rd basal FCM sample	08:00 AM (24 h after gastroscopy of each horse) gastroscopy FCM sample

AM: ante meridian; FCM: fecal cortisol metabolites; SC: salivary cortisol.

**Table 2:** Clinical examination values (temperature, respiratory rate, heart rate) of the horses participating in the first (N = 31) and second study period (N = 31).

Clinical Parameter	1st Study Period: Median (25th–75th Percentile)	2nd Study Period: Median (25th–75th Percentile)
Temperature °C Respiratory rate per minute Heart rate bpm	37.7 (37.3–37.7) 10 (8–12) 40 (36–42)	37.5 (37.3–37.7) 12 (10–12) 40 (36–40)

**Table 3:** Salivary cortisol values in young Thoroughbred racehorses before and after gastroscopy.

Salivary Cortisol Concentrations ng/ml	N	1st Study Period: Median (25th–75th Percentile)	N	2nd Study Period: Median (25th–75th Percentile)
Basal SC concentration(ng/mL) SC concentration immediately after gastroscopy (ng/mL) SC concentration 30 min after gastroscopy (ng/mL)	24 24 22	1.8 (1.1–3.6) ^a^ 3.4 (2.3–4.5) ^b^ 3.2 (2.7–3.9) ^b^	31 30 30	1.8 (1–2.3) ^c^ 3.7 (1.9–6) ^d^ 2.7 (1.5–3.9) ^e^

N: number of horses; ^a,b,c,d,e^ values with different letters within columns differ significantly.

**Table 4:** Fecal cortisol metabolites values (N = 31) in young Thoroughbred racehorses before and after gastroscopy.

Fecal Cortisol Metabolites Concentrations ng/g	1st Study Period: Median (25th–75th Percentile)	2nd Study Period: Median (25th–75th Percentile)
Basal FCM concentrations (ng/g) Gastroscopy FCM concentrations (ng/g)	9.4 (7.9–11.2) ^a^ 12.1 (9.6–16.3) ^b^	7.8 (6.4–9.1) ^c^ 10.1 (6.8–15.9) ^d^

N: number of horses, ^a,b,c,d^ values with different letters within columns differ significantly.

## Data Availability

The original contributions presented in the study are included in the article and Supplementary Materials, further inquiries can be directed to the corresponding author.

## Supplementary Materials

The following supporting information can be downloaded at: https://www.mdpi.com/article/10.3390/ani14223332/s1, Table S1: Hematology and serum biochemistry of the two-year-old Thoroughbreds in the first and second examination period.
