# Supplementary material for: Effects of a Gastroscopic Procedure on Salivary Cortisol Release and Fecal Cortisol Metabolites in Young Racehorses
Source: Animals (Basel). 2024 Nov 19;14(22):3332. doi: 10.3390/ani14223332 (PMC11590924; doi:10.3390/ani14223332)
Supplement: Supplementary file 1 [file animals-14-03332-s001.zip › animals-3275864-supplementary.pdf]

Supplementary Materials:

Table S1. Hematology and serum biochemistry of the two-year-old Thoroughbreds in the first and second examination period.

Parameter	N	1st study period: median (25th–75th percentile)	N	2nd study period: median (25th–75th percentile)
White blood cells (G/L)	31	8.3 (6.9–9.2)	30	6.8 (5.9–8.3)
Red blood cells (T/L)	31	9.4 (8.9–10.1)	30	8.7 (8.1-9.8)
Hemoglobin (G/L)	31	141 (129–147)	30	132 (124-146)
PCV (L/L)	31	0.4 (0.4–0.4)	30	0.4 (0.3-0.4)
MCV (fL)	31	40.2 (38.7–41)	30	42.1 (41-43)
MCH (pg)	31	14.7 (14.4–15.1)	30	15.2 (14.9-15.5)
MCHC (g/dL)	31	36.6 (36.2–37.5)	30	36.2 (35.6-36.8)
Platelet count (G/L)	31	144 (122–158)	30	134 (114-145)
MPV (fL)	31	7.1 (6.4–8.5)	30	6.2 (6-6.8)
Lymphocytes (%)	31	42.2 (39.7-45.8)	30	38.7 (33.7-42.6)
Monozyten (%)	31	3.0 (2.7–3.4)	30	3.4 (2.7-4.1)
Eosinophils (%)	31	1.4 (1.1-2)	30	1.7 (1.1-3)
Total protein (g/L)	31	59.9 (57.3–64.2)	27	58.6 (56.7-61.4)
Albumin (g/L)	31	35.1 (33.9–35.6)	29	34.4 (32.2-35.6)
GLDH (IU/L)	31	2.9 (1.8–5.8)	29	2.1 (1.9–3.2)
ALP (IU/L)	31	142 (127–155)	29	113 (102-127)
AST (IU/L)	31	208 (177–335)	29	181 (167-242)
Gamma-GT (IU/L)	31	16 (11–24.5)	29	13 (9-18)
CK (IU/L)	31	120 (106–170)	29	115 (97.5-132)
LDH (IU/L)	31	177 (150–217)	29	163 (132-188)
Total bilirubin (μmol/L)	31	33.5 (25.8–40.6)	29	37.1 (28.1/48.9)
Bile acid (μmol/L)	31	5.4 (4.3–7.6)	29	5.3 (4.3-6.8)
Urea (mmol/L)	31	4.8 (4–5.2)	29	4.5 (4–5)
Creatinine (μmol/L)	31	111 (96.4–125)	29	115 (104-121)

PCV: packed cell volume, MCV: mean corpuscular volume, MCH: mean corpuscular hemoglobin, MCHC: mean corpuscular hemoglobin concentration, MPV: mean platelet volume, GLDH: glutamate dehydrogenase, ALP: alkaline phosphatase, AST: aspartate aminotransferase, gamma-GT: gamma-glutamyltransferase, CK: creatine kinase, LDH: lactate dehydrogenase.
